# Peanut Shell Extract and Luteolin Regulate Lipid Metabolism and Induce Browning in 3T3-L1 Adipocytes

**DOI:** 10.3390/foods11172696

**Published:** 2022-09-03

**Authors:** Wenrui Liu, Lihua Wang, Jie Zhang

**Affiliations:** Institute of Food Science and Technology, Chinese Academy of Agricultural Sciences, Beijing 100193, China

**Keywords:** anti-obesity, adipogenesis, AMPK, lipolysis, UCP1

## Abstract

Peanut shells are agricultural waste products that require utilization. The freeze-dried ethanolic peanut shell extract (PSE) contained 10.01 ± 0.55 mg/g of luteolin (LUT) with a total polyphenol content of 18.11 ± 0.88 mg GAE/g. Thus, LUT is one of the major polyphenolic components in PSE. Although PSE displays antibacterial and neurotrophic activities, minimal research is available addressing its potential role in lipid metabolism. This study investigated the role of PSE in terms of inhibiting adipogenesis, accelerating lipolysis, and promoting lipid browning using the 3T3-L1 cell line. Without affecting cell viability, high concentrations of PSE and LUT prevented adipogenesis by reducing the mRNA levels of C/EBPα, PPARγ, and SREBP1-c, and increasing the protein levels of pACC and pAMPK. Moreover, PSE and LUT induced lipolysis by activating lipolytic proteins, and enhanced the protein expressions of the brown adipocyte-specific markers, UCP1, PGC-1α, and SIRT1 in fully differentiated 3T3-L1 adipocytes. Increased mitochondrial biosynthesis provided additional evidence in favor of these findings. Due to their anti-obesity properties, it is proposed that PSE and LUT could be used as potential dietary supplements.

## 1. Introduction

Obesity, caused by an imbalance in energy storage and expenditure, has become a serious public health challenge and is closely related to the development of chronic illnesses such as type 2 diabetes and cardiovascular disease [1,2]. Adipocyte differentiation determines, to a great extent, the increase in adipose tissue mass in obesity, which is characterized by an increase in the number and size of the adipocytes. However, lipolysis is one of the most effective ways of reducing lipid accumulation, leading to the degradation of triglycerides and the release of fatty acids [3]. Therefore, strategies aimed at regulating lipid metabolism and thus reducing lipid accumulation may be beneficial in preventing obesity and obesity-related chronic diseases. The currently known anti-obesity effects of phytochemical constituents highlight several possible mechanisms of lipid metabolism. These mechanisms include: (1) The inhibition of lipid synthetase activity. For example, polyphenols in grape and pomegranate peels reduced FAS expression in 3T3-L1 adipocytes, and inhibit lipid synthesis [4,5]. (2) Increased lipolysis enzyme activity. The study showed that resveratrol and citrus polyphenols could increase the β-oxidation of fatty acids in mitochondria by regulating the activity of HSL and CPT-1, accelerating lipid degradation and reducing lipid accumulation [6]. The bioactivity of tea polyphenols to promote lipolysis and reduce weight has received more widespread attention, such as Fu-brick tea [7], Pu-erh tea [8], etc. (3) Stimulation of body heat production and acceleration of energy expenditure. Quercetin-rich onion skin extract can promote brown adipose-specific genes (SIRT1, PGC-1α, UCP-1), induce the browning of white adipose tissue, and increase energy expenditure [9]. Remarkably, polyphenols can often act synergistically to produce anti-obesity effects through multiple intracellular mechanisms.

Premature adipocytes are converted into mature adipocytes through a process known as adipogenesis, which is characterized by lipid droplet formation as well as modifications of gene expression and cellular morphology [10]. Some transcription factors directly affect the growth of adipocytes, such as sterol regulatory element-binding protein1-c (SREBP1-c), CCAAT/enhancer-binding protein *α* (C/EBP*α*), and peroxisome proliferator-activated receptor *γ* (PPAR*γ*) [11]. These elements induce proteins and genes that determine intracellular lipid accumulation, including adipocyte fatty acid binding protein (aP2), acetyl-CoA carboxylase (ACC), and fatty acid synthase (FAS) [12]. In addition, lipolysis is a consecutive process involving three different lipases: adipose triglyceride lipase (ATGL), hormone-sensitive lipase (HSL), and monoacylglycerol lipase (MGL). Lipid degradation releases free glycerol and fatty acids, decreasing adipocyte lipids [13]. Lipolytic enzymes are not the only determinants during lipid decomposition. As a protective protein on the surface of lipid droplets, Perilipin A plays a key role in preventing the degradation of triglycerides, which is known as the “molecular switch” of lipid metabolism regulation [14]. After some stimulation, under the catalysis of Perilipin A, HSL is more easily transferred to the surface of lipid droplets and promotes lipolysis, while inhibiting lipolysis by limiting lipase binding to lipid droplets in basal conditions.

There are two main types of adipose tissue in mammals. The main function of white adipose tissue (WAT) is responsible for storing energy in the form of triglycerides, which are stored in unilocular lipid droplets, while brown adipose tissue (BAT) consists of brown adipocytes, which are characterized by multilocular lipid droplets and abundant mitochondria that dissipate energy. This leads to uncoupled respiration, which in turn causes heat generation (non-shivering thermogenesis) [15]. Increased mitochondrial numbers and reduced lipid droplet sizes can coexist in adipocytes, which resemble the brown adipocyte phenotype, also known as beige adipose. Browning refers to the transition from WAT to beige adipose [16]. Many studies have shown that browning increases energy consumption and regulates lipid metabolism [17]. This phenomenon is marked by the high expression of uncoupling protein 1 (UCP1) and the activation of β-oxidation [18,19]. UCP1 disperses the buildup of H atoms inside the mitochondrial membrane, which causes thermogenesis rather than ATP synthesis [20]. These beige adipose cells can be induced by specific stimuli, such as exposure to cold or β-adrenergic receptor activation [21]. In addition to UCP1, recent studies have identified specific markers for browning, including PRDM16, SIRT1, and PGC-1, which are significantly affected by mitochondrial biogenesis [22,23,24]. To date, considerable work has gone into finding substances that induce browning, as well as uncovering regulatory lipid browning factors [25,26].

It has been estimated that 230–300 g of peanut shell are produced for every kg of peanuts, and 10.7–14 million tons of peanut shell waste were produced globally in the years 2017 and 2018 [27]; as a multi-purpose plant resource, its development and use in China remain minimal. In addition to a small part used for wood-based panel production and animal feed, the majority is used as fuel or waste residue and discarded, wasting an abundant natural resource and directly affecting the practical application value of peanut shells [28]. Peanut shells contain a large number of polyphenols, while the main active ingredient, luteolin (LUT), has been extensively studied due to its broad range of functional activities [29], including anti-inflammatory, anti-allergic, and anti-diabetic properties [30,31,32]. Studies have demonstrated that LUT can inhibit pre-adipocyte differentiation and effectively ameliorate obesity [33]. It may also reduce insulin (INS) resistance by activating AMPK signaling in animal studies to reduce obesity [34]. This study aims to clarify the functional roles of peanut shell extract (PSE) and its major active compound LUT in lipid metabolism events and WAT browning in 3T3-L1 adipocytes.

## 2. Materials and Methods

### 2.1. Preparation of the PSE

The PSE was prepared using a previously described procedure with some modifications [29]. Fresh peanuts were purchased from a supermarket in Beijing, China. The peanuts were washed, dried, shelled, and crushed before being sonicated in 70% ethanol for 40 min. The extraction cycle was repeated three times. The ethanol extract was filtered by centrifugation and evaporated to 1/4 of the original volume using a rotary evaporator at 35 °C. The concentrated solution was lyophilized with a freeze dryer system to obtain the PSE powders. The total phenolic content in the PSE was determined via a Folin–Ciocalteu assay and expressed as mg/L gallic acid equivalents (GAE) [35].

### 2.2. Cell Culture and Treatment

Dulbecco’s Modified Eagle’s Medium (DMEM), Thermo Fisher Scientific (Waltham, MA, USA), supplemented with 10% newborn calf serum (NCS, Tianhang, Hangzhou, China) and 100 mg/mL of penicillin–streptomycin (P/S, Thermo Fisher Scientific, Waltham, MA, USA), was used to culture 3T3-L1 pre-adipocytes (ATCC-CL173, Manassas, VA, USA) at 37 °C in a 5% CO_2_ incubator until confluence. Then, 2 d after 100% confluence (0 d), the medium was replaced with a differentiation medium containing 10% fetal bovine serum (FBS, Tianhang, Hangzhou, China), 1% P/S, 0.5 mM 3-isobutyl-1-methylxanthine (IBMX, Yuanye, Shanghai, China), 10 μg/mL INS (Yuanye, Shanghai, China), and 1 μM dexamethasone (Dex, Yuanye, Shanghai, China). After 2 d, the cells were cultured in DMEM supplemented with 10% FBS, 1% P/S, and 10 μg/mL INS for an additional 2 d. At 5 d, the medium was replaced with a fresh medium containing DMEM, 10% FBS, and 1% P/S, which was changed every 48 h until 10 d. To evaluate the inhibitory effect, the confluent pre-adipocytes were incubated with various PSE and LUT (Yuanye, Shanghai, China) concentrations until the formation of fully differential adipocytes, which were used for different analyses.

### 2.3. Cell Viability Assay

After 3T3-L1 pre-adipocyte proliferation to more than 80% confluency, the cells were cultured with different PSE and LUT concentrations (5–40 mg GAE/L) for 48 h. The DMSO-treated (1:1000) pre-adipocytes were used as the control. After the completion of drug intervention, incubation was continued for 4 h after adding 20 μL of 3 mg/mL MTT to each well. Next, the culture medium was removed, and 200 mL DMSO was added. The absorbance at 490 nm was measured by a microplate reader after the plate was kept at room temperature for 30 min.

### 2.4. Oil Red O Staining

The intracellular lipid accumulation was determined via Oil Red O staining [36]. First, the cultured adipocytes were gently washed twice with phosphate-buffered saline (PBS, pH 7.4), then fixed at room temperature in 4% paraformaldehyde for 45 min and washed with 60% isopropanol. Then, the cells were stained for 20 min with a solution of 0.6% Oil Red O dye, then washed four times with deionized water, after which images were captured using a microscope. To quantitatively analyze the staining results, the Oil Red O dye was removed and then added to absolute ethyl alcohol for 30 min to dissolve the bound staining. The dissolved Oil Red O content was examined using a microplate reader to measure the absorbance at 510 nm.

### 2.5. Lipolysis Assay

The lipolysis was assessed by measuring the amount of glycerol released by the cells in the medium [37]. The fully differentiated 3T3-L1 adipocytes were serum-starved for 12 h (The medium did not contain NCS) and then treated with various concentrations of PSE and LUT for 24 h and 48 h in phenol red-free DMEM. The supernatant of the medium was collected for a glycerol release assay using a glycerol assay kit (APPLYGEN, Beijing, China).

### 2.6. Intracellular ATP Determination

At 10 d after 3T3-L1 pre-adipocyte differentiation, the medium was removed, and the adipocytes were incubated with a medium containing PES and LUT for 48 h. The intracellular ATP levels were determined using an ATP Content Assay Kit (Solarbio, Beijing, China) following the instructions of the manufacturer. Briefly, the glucose and ATP were catalyzed by hexokinase to produce glucose 6-phosphate, which was further dehydrogenated to produce NADPH. The NADPH content was determined using a microplate reader to measure the absorbance at 340 nm. The amount of ATP was proportional to that of NADPH.

### 2.7. Mitochondrial Staining

After the cells were incubated with different PSE and LUT concentrations, the medium was replaced with PBS containing 100 nM Mito Tracker Green and the differentiated adipocytes were incubated at 37 °C in the dark for 30 min. After incubation, the cells were slightly washed with PBS to remove unbound dyes, after which the mitochondria displayed green fluorescence when observed under the fluorescence microscope.

### 2.8. RNA Extraction and Real-Time Quantitative PCR (RT-qPCR)

The total RNA was extracted from the 3T3-L1 adipocytes treated with PSE and LUT using Trizol reagent (Gene-Protein Link, Beijing, China), and quantified spectrophotometrically. Then, cDNA was synthesized using a cDNA reverse transcription kit (TransGen Biotech, Beijing, China), with 1 μg of RNA used for reverse transcription, according to the instructions of the manufacturer. The mRNA expression level was measured via quantitative real-time PCR using SYBER Green PCR Master Mix (TransGen Biotech, Beijing, China) and a 7500 Real-Time PCR system (Applied Biosystems Foster City, CA, USA) according to the instructions of the manufacturer. The 13 pair primers of targeted genes were designed by Primer Premier 6.0 software (PREMIER Biosoft, Palo Alto, CA, USA) and are shown in Table 1. Primers were synthesized by Beijing Tsingke Biotechnology Co., Ltd. (Beijing, China). The relative mRNA expression was analyzed using the 2^−ΔΔCT^ method with β-actin. All RT-qPCR analyses were repeated three times.

### 2.9. Western Blot Analysis

The total protein was collected in the cells at different times using RIPA Buffer (Beyotime Biotechnology, Shanghai, China). After centrifugation (12,000× g, 15 min), the protein concentration in the supernatant was measured using BCA protein assay kits. An equal amount of each sample was loaded and separated in 10% acrylamide SDS/PAGE and transferred to a polyvinylidene fluoride (PVDF) membrane (Gene-Protein Link, Beijing, China). Then, the membranes were blocked in 5% non-fat skimmed milk at room temperature for 1 h and incubated with primary and secondary antibodies. The primary antibodies used in this study were anti-β-actin, anti-p-AMPK (Thr172), anti-AMPK, anti-ACC, anti-p-ACC, anti-HSL, anti-p-HSL, anti-UCP1, anti-PGC-1α, and anti-Sirt1. After washing three times with TBST, the proteins were visualized using enhanced chemiluminescence (Gene-Protein Link, Beijing, China) in a ChemiDoc XRS System (Bio-Rad, Shanghai, China). The densities of the protein bands were normalized to β-actin bands and quantified using ImageJ software (National Institutes of Health, Bethesda, MD, USA). All data were expressed as the densities of the protein bands using ImageJ software, which was normalized to β-actin and expressed as a percentage of the control.

### 2.10. Statistical Analysis

All data were expressed as the mean ± standard error of the mean (SEM). The statistical analysis was performed via one-way analysis of variance (ANOVA) and Tukey’s multiple comparison tests using GraphPad Prism 8 software (GraphPad, San Diego, CA, USA). For all data comparison analyses, *p*-values of less than 0.05 were considered to be statistically significant.

## 3. Results

### 3.1. The Effect of PSE and LUT on Cell Viability

**Figure 1:**
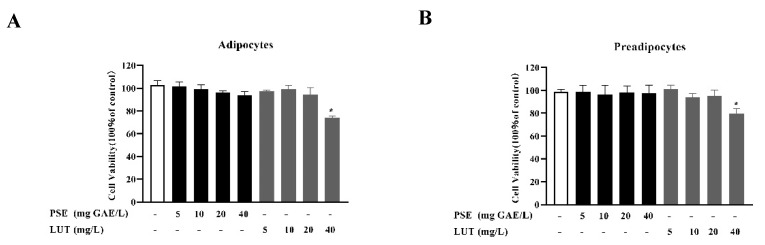
The cell viability of the 3T3-L1 cells in the (**A**) preadipocytes and (**B**) adipocytes treated with PSE and LUT or a vehicle 48 h after cell plating. Data are presented as mean ± SD from three independent experiments. * Significant differences are indicated by *p* < 0.05.

The cytotoxicity of PSE and LUT to 3T3-L1 pre-adipocytes and mature adipocytes were investigated at different doses (5–40 mg/L). As shown in Figure 1, treatment with PSE at concentrations of up to 40 mg/L displayed no significant cytotoxic effect on the 3T3-L1 pre-adipocytes and mature adipocytes compared with the control group (1:1000 DMSO treatment). However, at 40 mg/L, LUT exhibited cytotoxicity in the 3T3-L1 pre-adipocytes and mature adipocytes. Therefore, 40 mg GAE/L, 20 mg GAE/L, 10 mg GAE/L PSE and 20 mg/L, 10 mg/L, and 5 mg/L LUT were chosen for the subsequent experiments.

### 3.2. The Effect of PSE and LUT on Intracellular Lipid Accumulation in Adipocytes

**Figure 2:**
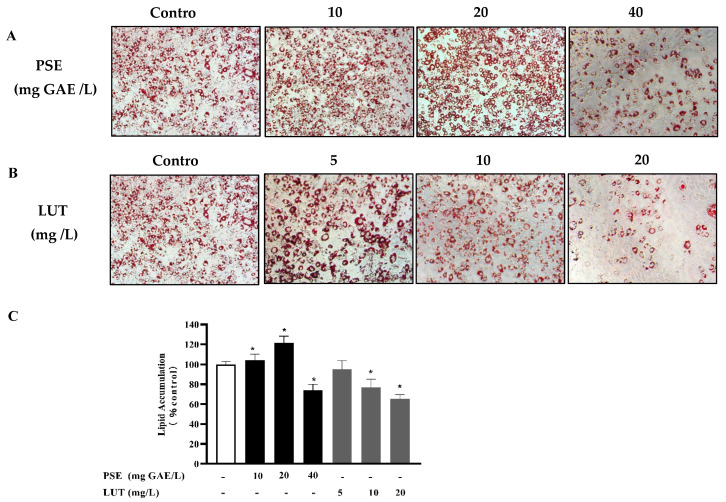
The effect of PSE and LUT on the lipid accumulation in 3T3-L1 adipocytes after 10 d. (**A**) Oil Red O staining of the 3T3-L1 cells treated with different PSE concentrations (0 mg GAE/L, 10 mg GAE/L, 20 mg GAE/L and 40 mg GAE/L) at 200× magnification. (**B**) Oil Red O staining of the 3T3-L1 cells treated with different LUT concentrations (0 mg/L, 5 mg/L, 10 mg/L and 20 mg/L) at 200× magnification. Scale bar = 200 μm. (**C**) Quantification by measuring the absorbance at 510 nm. Data are presented as the mean ± S.D. from three independent experiments. * Significant differences are indicated by *p* < 0.05.

The effect of PSE and LUT on lipid accumulation in 3T3-L1 adipocytes was measured using Oil Red O staining. As shown in Figure 2A,C, the modulatory effect of PSE on adipogenesis was biphasic. At lower concentrations of 10 mg GAE/L and 20 mg GAE/L, the PSE significantly promoted lipid accumulation, while the opposite was evident at 40 mg GAE/L. As shown in Figure 2B,C, intracellular lipid accumulation significantly decreased in a dose-dependent manner in the LUT-treated groups. Furthermore, 5 mg/L LUT had no significant inhibitory effect on lipid accumulation. Concentrations of up to 10 mg/L of LUT resulted in a significant lipid accumulation effect compared with the control. Lipid accumulation was reduced by 22.9% and 34.6%, respectively, at 10 mg/L and 20 mg/L LUT.

### 3.3. PSE and LUT Modulated the Expressions Levels of mRNA and Protein Involved in AdipoGenesis

**Figure 3:**
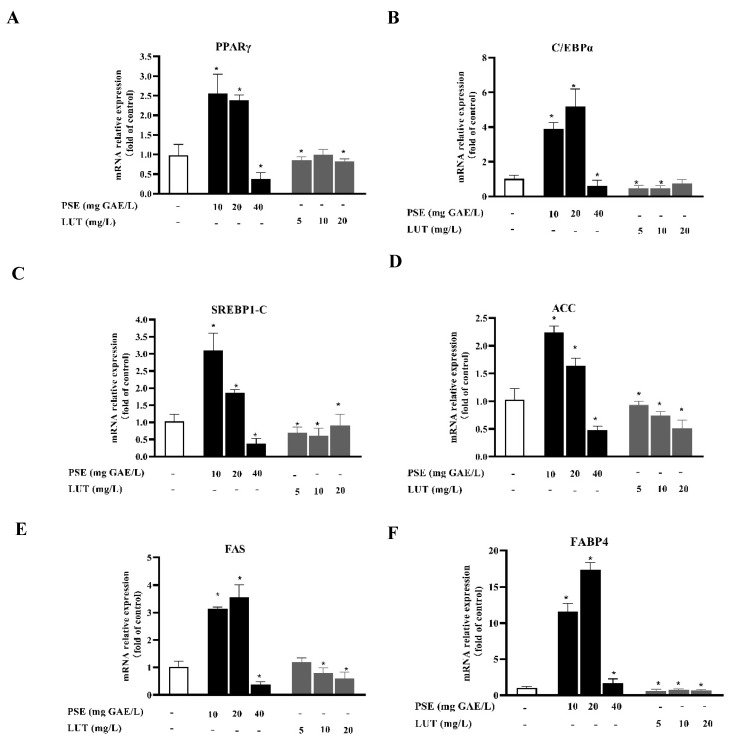
PSE and LUT modulated the expression of mRNA involved in adipogenesis in the 3T3-L1 adipocytes. The relative mRNA expressions of (**A**) PPARγ, (**B**) C/EBPα, (**C**) SREBP1-C, (**D**) ACC, (**E**) FAS, and (**F**) FABP4. The data are presented as the mean ± S.D. from three independent experiments. * Significant differences are indicated by *p* < 0.05.

To determine whether PSE and LUT affected adipogenesis at the molecular level, western blotting and qPCR analysis were performed to measure the related protein and mRNA transcription levels. PPARγ, C/EBPα, and SREBP1-C are the three key adipogenic transcription factors that trigger the expression of a large number of downstream adipogenic genes (ACC, FAS, and FABP4) when the adipocyte differentiation process enters its final stage. As shown in Figure 3, the mRNA levels of PPAR-γ, C/EBP-α, SREBP-1c, ACC, FAS, and FABP4 decreased by 62.3%, 39.8%, 61.23, 51.8%, 62.7%, and 31.5%, respectively, after 40 mg GAE/L PSE treatment compared with the control group. Contrarily, the mRNA expression levels of these genes significantly increased after treatment at concentrations of 10 mg GAE/L and 20 mg GAE/L, compared with the control cells. The results verify that the effect of PSE on lipid accumulation was dual. Treatment with various LUT concentrations significantly downregulated the gene expression involved in adipogenesis. Contrary to the expected experimental results, the expression of these genes was not dose-dependent.

**Figure 4:**
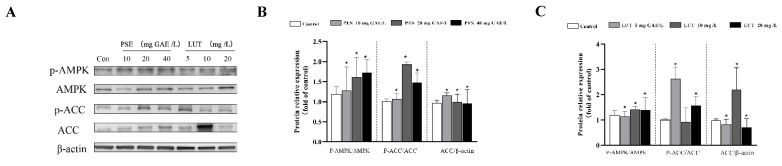
PSE and LUT modulated the expression of the phosphorylation of AMPK and ACC in the 3T3-L1 adipocytes. (**A**) Western blot results for the phosphorylation of ACC and AMPK. Quantification of pACC/ACC, pAMPK/AMPK, and ACC/β-actin after (**B**) PSE and (**C**) LUT treatments. The data are presented as the mean ± SD from three independent experiments. * Significant differences are indicated by *p* < 0.05.

AMPK plays a central role in regulating lipid metabolism in the adipose tissue. As shown in Figure 4B, we measured the protein levels of phosphorylated AMPK and its substrate, ACC, by Western blot. Our results indicate that PSE intervention significantly increased the AMPK phosphorylation level compared with the control group. In total, 10, 20 and 40 mg/L PSE increased AMPK phosphorylation by 6%, 35% and 44%, respectively. In addition, AMPK phosphorylation induces phosphorylation of the downstream protein ACC, which leads to its inactivation and down-regulates ACC protein expression. These results indicate that PSE activates the AMPK signaling pathway and inhibits adipogenesis. In the LUT-treated groups, as shown in Figure 4C, LUT also remarkably enhanced the phosphorylation of AMPK. Although the phosphorylated expression of downstream protein ACC fluctuated under different concentrations of the LUT treatment, the results are similar to those of the PSE treatment at higher concentrations of LUT treatment.

### 3.4. The Effect of PSE and LUT on the Lipolysis in Differentiated 3T3-L1 Adipocytes

**Figure 5:**
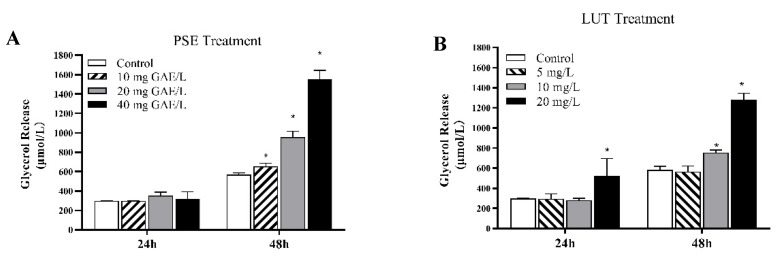
The effect of PSE and LUT on adipocyte lipolysis. Fully differentiated adipocytes treated with various concentrations of (**A**) PSE (0 mg GAE/L, 10 mg GAE/L, 20 mg GAE/L and 40 mg GAE/L) and (**B**) LUT (0 mg/L, 5 mg/L, 10 mg/L and 20 mg/L) for 24 h and 48 h, respectively. The data are presented as the mean ± SD from three independent experiments. * Significant differences are indicated by *p* < 0.05.

Triacylglycerol hydrolysis proportionally releases glycerol and free fatty acid from adipocytes. The glycerol release in the supernatant of the medium was evaluated as an index of lipolysis. The 3T3-L1 adipocytes were incubated with PSE and LUT at the indicated concentrations for 24 h and 48 h. As shown in Figure 5, the glycerol release showed a time-dependent response, in which the glycerol released into the supernatant of the 48 h treatment group was higher than that of the 24 h treatment group in both the PSE- and LUT-treated groups. As shown in Figure 5A, after 24 h of PSE treatment, no significant differences were evident between the glycerol levels of the medium supernatant of the PSE-treated and control groups. However, incubation with 10 mg GAE/L, 20 mg GAE/L, and 40 mg GAE/L PSE for 48 h increased the glycerol release by 14.9%, 67.9%, and 170%, respectively. As shown in Figure 5B, after 24 h of LUT treatment, only the 20 mg/L LUT group showed a significant difference in glycerol release compared with the control group, and the other groups had no glycerol release, similar to the PSE group. Here, 10 mg/L and 20 mg/L LUT significantly increased the glycerol content by 29.3% and 119%, while 5 mg/L LUT had no effect on the release of glycerol after 48 h of LUT treatment.

### 3.5. PSE and LUT Modulated the Expression Levels of mRNA and Protein Involved in Lipolysis

**Figure 6:**
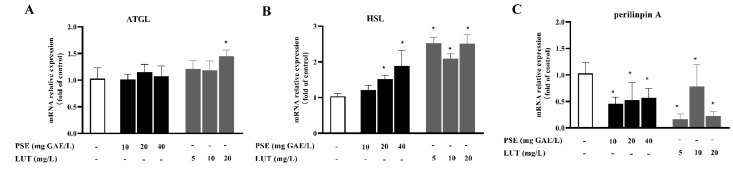
PSE and LUT modulated the expression of the mRNA involved in the lipolysis of the 3T3-L1 adipocytes. The relative mRNA expression of (**A**) ATGL, (**B**) HSL, and (**C**) Perilinpin A. The data are presented as the mean ± SD from three independent experiments. * Significant differences are indicated by *p* < 0.05.

To determine the impact of PSE and LUT on lipolysis at the molecular level, western blotting and qPCR analysis were performed to measure the related mRNA transcription and protein levels. ATGL and HSL are responsible for breaking down triacylglycerol, while Perilipin A is believed to regulate lipolysis by acting as a barrier to restrict lipase access to the lipid droplet. As shown in Figure 6A, the gene expression of ATGL was not affected by PSE and lower doses of LUT, while the ATGL expression was increased in the differentiated 3T3-L1 cells by 20 mg/L LUT treatment. As shown in Figure 6B,C, PSE and LUT treatment increased the HSL gene levels and decreased the Perilipin A gene levels.

**Figure 7:**
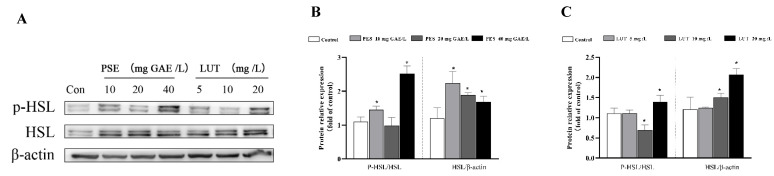
PSE and LUT modulated the expression of the phosphorylation of HSL in the 3T3-L1 adipocytes. (**A**) The Western blot results for the HSL phosphorylation. The quantification of pHSL/HSL and HSL/β-actin after (**B**) PSE and (**C**) LUT treatment. The data are presented as the mean ± SD from three independent experiments. * Significant differences are indicated by *p* < 0.05.

HSL is the rate-limiting enzyme for lipid hydrolysis, and HSL phosphorylation plays a key role in the lipolysis process. As shown in Figure 7A, we measured the protein levels of phosphorylated HSL using Western blot. The results indicate that 20 mg GAE/L PSE and 5 mg/L LUT did not significantly promote the phosphorylation of HSL, and 40 mg/L PSE and 20 mg/L LUT significantly increased HSL phosphorylation by 127.6% and 30.5%, respectively. Interestingly, it is estimated that there is a dose–effect relationship between LUT concentration and HSL phosphorylation expression. The expression levels of HSL protein increased by 85.2%, 30.3% and 25.5%, respectively, after 10, 20 and 40 mg GAE/L PSE treatment. The protein expression of HSL was also increased to varying degrees in LUT treatment.

### 3.6. The Effect of PSE and LUT on Mitochondrial Biogenesis

**Figure 8:**
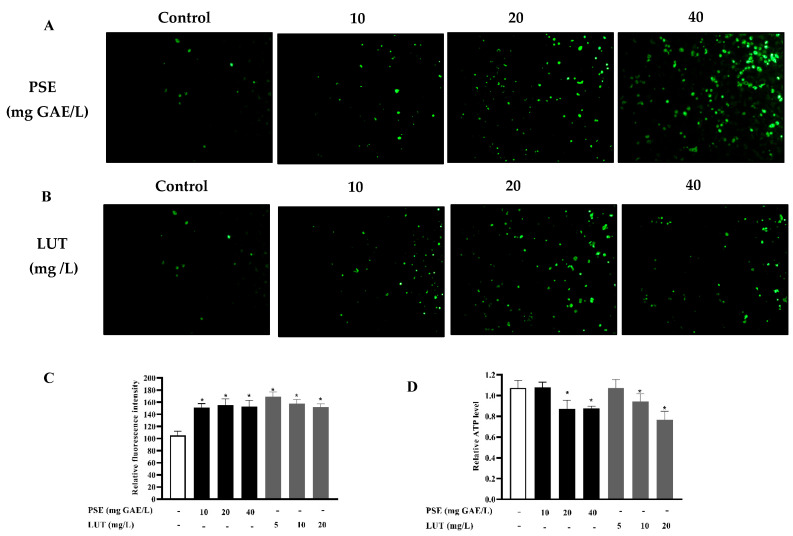
The effect of PSE and LUT on the mitochondrial biogenesis of fully differentiated 3T3-L1 adipocytes after 48 h. (**A**) Mito Tracker Green staining of fully differentiated 3T3-L1 adipocytes treated with different PSE concentrations (0 mg/L, 10 mg/L, 20 mg/L and 40 mg/L) at 200× magnification. (**B**) Mito Tracker Green staining of fully differentiated 3T3-L1 adipocytes treated with different LUT concentrations (0 mg/L, 5 mg/L, 10 mg/L and 20 mg/L) at 200× magnification. Scale bar = 200 μm. (**C**) Quantified via the fluorescence intensity measuring of the mitochondrial mass. (**D**) The intracellular ATP level. The data are presented as the mean ± SD from three independent experiments. * Significant differences are indicated by *p* < 0.05.

Next, this study explored whether PSE and LUT increased the mitochondrial mass and function in a manner that induces browning, which is closely related to lipolysis. Fully differentiated 3T3-L1 adipocytes were treated with PSE and LUT for 48 h, after which the mitochondrial mass was determined via fluorescence quantification using Mito Tracker. The level of intracellular ATP was also measured to further evaluate the mitochondrial functionality. As shown in Figure 8C, the quantitative analysis indicated that PSE and LUT treatment significantly increased the mitochondrial fluorescence intensity compared with the control group. PSE (10 mg GAE/L, 20 mg GAE/L, and 40 mg GAE/L) significantly increased the mitochondrial mass by 43.78%, 47.81%, and 45.51%, respectively, while LUT (5 mg/L, 10 mg/L, and 20 mg/L) facilitated increases of 61.12%, 50.23%, and 44.81%. As shown in Figure 8D, PSE and LUT both decreased the ATP levels.

### 3.7. PSE and LUT Modulated the Expression Levels of the mRNA and Protein Involved in Browning

**Figure 9:**
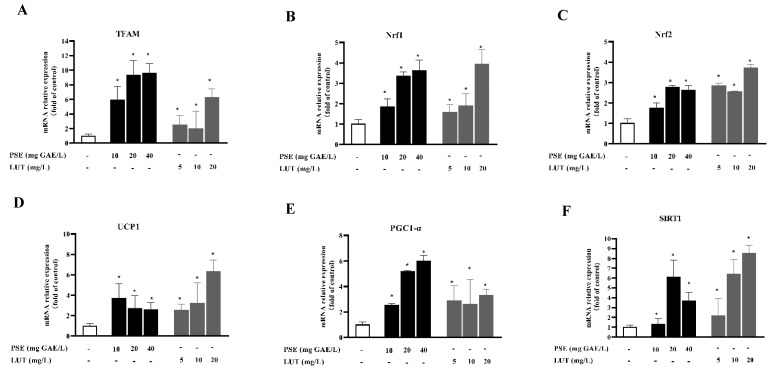
PSE and LUT modulated the expression of the core browning gene in the 3T3-L1 adipocytes. The relative mRNA expressions of (**A**) TFAM, (**B**) Nrf1, (**C**) Nrf2, (**D**) UCP1, (**E**) PGC1-α, and (**F**) SIRT1. The data are presented as the mean ± SD from three independent experiments. * Significant differences are indicated by *p* < 0.05.

**Figure 10:**
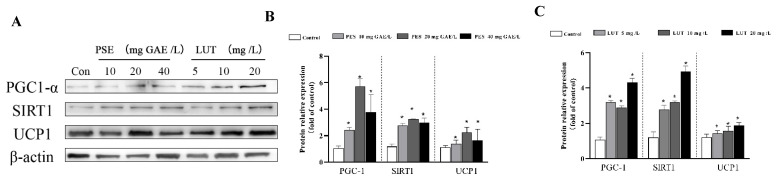
PSE and LUT modulated the expression of core browning protein markers. (**A**) Western blot results for the browning proteins. The quantification of PGC1-α, SIRT1, UCP1 after (**B**) PSE and (**C**) LUT treatment. The data are presented as the mean ± SD from three independent experiments. * Significant differences are indicated by *p* < 0.05.

Brown adipocytes are characterized by a high level of UCP1 expression and increased mitochondrial content and energy homeostasis. To investigate the browning effect of PSE and LUT, the fully differentiated 3T3-L1 adipocytes were treated with different concentrations of PSE and LUT for 48 h while determining the expression levels of the core browning gene and protein markers, including UCP1, PGC-1α, and SIRT1. As shown in Figure 9 and Figure 10, the PSE and LUT treatment significantly upregulated these BAT-specific genes and proteins compared with the control. The enhanced expression of these BAT markers suggests the possible conversion of WAT browning. In addition, the PSE and LUT treatments markedly increased the Tfam, Nrf1, and Nrf2 gene expression levels, while these genes were responsible for mitochondrial biogenesis.

## 4. Discussion

Adipocytes can store triglycerides or release fatty acids, which are crucial for maintaining lipid homeostasis and maintaining a healthy energy balance [38]. When energy intake exceeds energy output, the excess energy is stored in the form of triglycerides in cells, promoting the accumulation of lipids in the body and leading to obesity [39]. Therefore, inhibiting adipogenesis and stimulating lipolysis is a promising approach for preventing and managing obesity. Additionally, the regulation of lipid accumulation is often accompanied by changes in thermogenesis, which is regulated by mitochondria as energy receptors [40]. LUT is a plant-derived flavonoid abundant in peanut shells [41]. LUT displays potent antioxidant activity and plays a variety of biological and pharmaceutical roles as secondary metabolites in plants [42]. Previous studies have shown that LUT can improve obesity-induced insulin resistance [43] and inhibit adipogenic differentiation [33]. This study demonstrated that both LUT and PSE inhibited adipogenesis in 3T3-L1 pre-adipocytes, while promoting lipolysis and mitochondrial biogenesis in fully differentiated 3T3-L1 adipocytes to decrease intracellular lipid accumulation. The potential regulatory mechanism of their anti-obesity effect was also investigated.

The formation of lipid droplets can be considered an important marker of the full differentiation of 3T3-L1 pre-adipocytes. Preventing lipid differentiation is one way to reduce lipid accumulation, consequently presenting an anti-obesity effect [44]. Furthermore, lipid accumulation was dramatically inhibited by LUT treatment in a dose-dependent manner, while PSE promoted lipid accumulation at low concentrations and inhibited lipid accumulation at high concentrations. During the lipid formation process, some transcription factors, as the starting switch of lipid accumulation, such as PPAR-γ, CEBP-α, and SREBP-1c, are expressed first and interact with each other to further induce the expression of FABP4, ACC1, and FAS, which are involved in lipid synthesis. In this study, LUT at all concentrations and PSE at high concentrations significantly down-regulated PPAR-γ, CEBP-α, SREBP-1c, FABP4, ACC1, and FAS in 3T3-L1 adipocytes, while PSE at low concentrations upregulated these genes. It is assumed that single compounds may more effectively inhibit lipid formation, because it is a highly refined type of matter. PSE is a mixture of substances containing not only LUT but also many other compounds, including carotene, saponin, xylose, and sitosterol [45]. The combined impacts of various substances may affect lipid accumulation differently from those of monomers. For example, the bioactive compound tilianin showed more remarkable effects than *Agastache rugosa* extract during lipid accumulation [46]. Compared to using a single flavone, when multiple flavones in the *Artemisia sacrorum* were combined, they markedly reduced lipid accumulation [47]. However, the AMPK is a key enzyme for energy regulation in cells and inhibits adipogenesis by blocking the expression of transcription factors in 3T3-L1 adipocytes [48]. This study indicates that PSE and LUT increased the phosphorylation levels of both AMPK and ACC in the 3T3-L1 adipocytes, which is similar to results in previous reports [49,50].

Lipolysis is a metabolic pathway that reduces lipid accumulation, playing a crucial role in the hydrolysis of triglycerides, and is critical to metabolic health. Minimal studies are available regarding the lipolysis activity of PSE on adipocytes in vitro. This study has revealed that a short duration of stimulation has a negligible impact on lipolysis (24 h), while PSE and LUT significantly affected lipolysis only when the stimulation duration was extended (48 h). Previous results show that proanthocyanidins induce dose- and time-dependent lipolysis in adipocytes, and that there is a synergistic effect [51], which is generally consistent with the results of the present study. ATGL and HSL are essential for intracellular triglyceride degradation and are commonly used in lipolysis studies [52]. In this study, the expression of ATGL was almost unaffected by PSE and LUT at the mRNA level, while the expression of HSL was markedly increased. In addition, consistent with the mRNA level, PSE and LUT substantially increased the HSL protein levels and phosphorylation. Furthermore, PSE and LUT treatment significantly decreased the mRNA expression of Perilipin A, a protein covering the large lipid droplets in adipocytes and an important lipid storage regulator [53]. Previously, many studies have found that a variety of polyphenol-rich plant extracts upregulate the expressions of these genes and proteins, and promote lipolysis in cell and animal models. For example, almond skin polyphenol extract promotes HSL phosphorylation and ATGL protein expression through AMPK pathway [54], and *allium hookeri* root extract promotes the mRNA expression of these lipases by the activation of PPAR-γ in WAT of HFD-induced mice [55]. The activation of PKA has been shown to play an important role in lipolytic regulation. When the PKA pathway is activated, HSL and Perilipin A are both phosphorylated. Phosphorylated HSL is transferred from the cytoplasm to the surface of the lipid droplet, consequently promoting triglyceride decomposition [56]. Phosphorylated Perilipin A can assist lipases in accessing the lipid droplet, eliminating the protective effect. The HSL and Perilipin A levels in mRNA are considered to be regulated in a coordinated manner during lipolysis, which is consistent with the study results. At the protein level, both HSL and ATGL phosphorylation are present during lipolysis, while ATGL phosphorylation is independent of PKA [57]. Therefore, it is postulated that the PSE- and LUT-induced lipolysis enhancement may be mostly dependent on the downregulation of Perilipin A and higher PKA-mediated HSL activation, since ATGL expression is not obvious at the mRNA level. In a study using ethanol extracts of *Smilax china L. leaf* to promote lipolysis, researchers measured the expression of upstream proteins of HSL, and clarified the activation of HSL phosphorylation by the PKA signaling pathway [58]. Besides the PKA signaling pathway, previous studies have shown that the activation of the ERK and AMPK pathways is also involved in regulating lipolysis. For instance, tumor necrosis factor-α-stimulated lipolysis in 3T3-L1 adipocytes depends on the downregulation of Perilipin A expression via ERK pathway activation [59]. The activation of the AMPK pathway can inhibit the movement of HSL to lipid droplets and limit the action of lipolytic enzymes [60]. Therefore, multiple pathway interactions require further investigation to further understand the mechanisms behind PSE and LUT lipolysis.

Beige adipocytes, which are dispersed in WAT, are characterized by an abundance of mitochondria and the high expression of UCP1. Mitochondria are important sites for cellular energy metabolism, and are capable of adaptive thermogenesis to convert chemical energy into thermal energy for dissipation and maintaining an energy balance in the body [61]. Mitochondrial biogenesis is a process that increases the functional activity of mitochondria and occurs in healthy cells, activated by a variety of signals involving energy stress and environmental stimuli [62]. This study found that PSE and LUT strongly impacted mitochondrial mass and function, increasing the mitochondrial content and decreasing cellular ATP levels, ultimately inducing WAT browning, which indicated the elevated expression of BAT-specific markers, UCP1, SIRT1, and PGC1-α at both the gene and protein levels. Various natural polyphenolic components were confirmed to induce browning and thermogenesis. For example, rutin upregulated the expression of UCP1 in BAT and enhanced muscle mitochondrial biogenesis through the AMPK pathway [63]. Furthermore, epicatechin reduces the rate of weight gain by regulating mitochondrial genesis-related proteins (Sirt-1, PGC1a) [64], suggesting that the supplementation of rutin or epicatechin could help avoid high-fat diet-induced obesity in mice. UCP1 is responsible for uncoupling the electron transport chain from energy production, resulting in the dissipation of biological energy that should be synthesized as ATP into heat energy [65]. The results show that the cellular ATP content was decreased in conjunction with an increase in the UCP1 expression level, and this result also occurred in the activation of mitochondrial function by coffee silverskin extracts [66] and sulforaphane [67]. This verifies that the decrease in ATP synthase may be a compensation for the increased expression of UCP1. Previous studies have confirmed the critical role of mitochondrial biosynthesis in beige adipocytes [68,69]. At the mRNA level, PSE and LUT significantly increased Tfam, Nrf1, and Nrf2 gene expression. These three genes are thought to be key biosynthetic markers in mitochondrial DNA replication and synthesis [70,71]. Previous studies have found that SIRT1 activates PGC-1α via deacetylation, and the further activation of NRF1 and TFAM, directly regulating mitochondrial DNA transcripton [72]. Nr2 activation upregulated the expression of NRF-1 and its downstream signaling molecules, and increased mitochondrial DNA copies, consequently participating in the regulation of mitochondrial biosynthesis [73]. It was reported that 6-Gingerol [74] and resveratrol [75] significantly increased the expression levels of mitochondrial biogenesis activator (Tfam and Nrf1) in 3T3-L1 cells, and improved mitochondrial functions, resulting in the browning transition of adipocytes. Therefore, both PSE and LUT promote mitochondrial biosynthesis in adipocytes, increase body energy metabolism, and induce lipid browning.

## 5. Conclusions

This study investigated the potential anti-obesity effect of PSE and LUT in both pre-adipocytes and fully differentiated adipocytes. In the adipocyte differentiation process, PSE and LUT regulate the expression of transcription factors (C/EBPα, PPARγ, and SREBP1-c) and lipid synthases (FABP4, ACC1, and FAS), resulting in the suppression of lipid accumulation. This process could depend on the activation of AMPK-mediated signaling pathways. Additionally, in fully differentiated adipocytes, PSE and LUT induced HSL phosphorylation and down-regulated Perilipin A gene expression, promoting lipolysis in differentiated adipocytes. Our findings also suggest that PSE and LUT increase mitochondrial biogenesis, accompanied by the expression of BAT-specific markers (UCP1, SIRT1, and PGC1-α). Based on the results of this study, it is considered that PSE and its major bioactive compound, LUT, may be used as a dietary food or supplement to address obesity. However, this study is still at the stage of in vitro cellular experiments, and many of the action mechanisms and the most relevant receptors in vivo remain unclear and require more extensive investigation.

## Figures and Tables

**Table 1:** Primer information.

Target Gene	Forward	Reverse
PPARγ	GACGCGGAAGAAGAGACCTG	GTGTGACTTCTCCTCAGCCC
C/ebp-α	AATGGCAGTGTGCACGTCTA	CCCCAGCCGTTAGTGAAGAG
SREBP1	CAGACTCACTGCTGCTGACA	GATGGTCCCTCCACTCACCA
ACC	GCCTCAGGAGGATTTGCTGT	AGGATCTACCCAGGCCACAT
FAS	GAGGGTGTGCCATTCTGTCA	GCTATTCTCTACCGCTGGGG
FABP4	GGATTTGGTCACCATCCGGT	TTCCATCCCACTTCTGCACC
HSL	AAAAGCTGAACCTGGGGGAG	TGCCATCTGCTGGGAAAACA
ATGL	AACGCCACTCACATCTACGG	TGGCAGGCATGGGACATAAA
Perilipin A	CTCAGCTCTCCTGTTAGGCG	GTAGAGCTCACCAAGGGCAG
UCP1	ACGTCCCCTGCCATTTACTG	GGTACGCTTGGGTACTGTCC
Ppargc1α	ACTCTCAGTAAGGGGCTGGT	ACATGTCCCAAGCCATCCAG
Sirt1	CGGCTACGAGGTCCATATAC	ACAATCTGCCACAGCGTCAT
β-actin	GAGCGCAAGTACTCTGTGTG	AACGCAGCTCAGTAACAGTC

## Data Availability

Data is contained within the article.
